# Maternal Hypothyroidism in Early Pregnancy and Infant Structural Congenital Malformations

**DOI:** 10.1155/2014/160780

**Published:** 2014-03-12

**Authors:** Bengt Källén, Birgitta Norstedt Wikner

**Affiliations:** ^1^Tornblad Institute, Lund University, Biskopsgatan 7, 223 62 Lund, Sweden; ^2^National Board of Health and Welfare, 10630 Stockholm, Sweden; ^3^Karolinska Institutet, Department of Medicine, Centre for Pharmacoepidemiology, 17176 Stockholm, Sweden

## Abstract

*Background*. The question is debated on whether maternal hypothyroidism or use of thyroxin in early pregnancy affects the risk for infant congenital malformations. *Objectives*. To expand the previously published study on maternal thyroxin use in early pregnancy and the risk for congenital malformations. *Methods*. Data from the Swedish Medical Birth Register were used for the years 1996–2011 and infant malformations were identified from national health registers. Women with preexisting diabetes or reporting the use of thyreostatics, anticonvulsants, or antihypertensives were excluded from analysis. Risk estimates were made as odds ratios (ORs) or risk ratios (RRs) after adjustment for year of delivery, maternal age, parity, smoking, and body mass index. *Results*. Among 23 259 infants whose mothers in early pregnancy used thyroxin, 730 had a major malformation; among all 1 567 736 infants, 48012 had such malformations. The adjusted OR was 1.06 (95% CI 0.98–1.14). For anal atresia the RR was 1.85 (95% CI 1.00–1.85) and for choanal atresia 3.14 (95% CI 1.26–6.47). The risk of some other malformations was also increased but statistical significance was not reached. *Conclusions*. Treated maternal hypothyroidism may be a weak risk factor for infant congenital malformations but an association with a few rare conditions is possible.

## 1. Introduction

Maternal hypothyroidism or hypothyroxinemia (normal TSH with low T4) has an effect on the neurodevelopment of also euthyroid infants [1, 2]. Less is known about a possible risk increase of structural congenital malformations, associated with maternal hypothyroidism or thyroxin use. A few case reports linked various malformations with maternal hypothyroidism (e.g., [3]). In the prospective Collaborative Perinatal Project [4], 537 pregnant women were identified who had used thyroxin in early pregnancy. There was a nonsignificant risk increase for any major malformation (1.26, 95% CI 0.91–1.75), notably for cardiovascular defects (1.72, 0.79–3.23) based on only 9 exposed cases.

A study from metropolitan Atlanta [5] found no overall relationship between maternal hypothyroidism or intake of thyroid drugs of thyroid drugs and the risk of infant congenital malformations but noted a doubling of the risk of infants with multiple malformations but these presented no uniform pattern. A study from Chile [6] found an odds ratio of 2.84 (95% CI 1.31–6.16) for any malformation after maternal hypothyroidism, about the same for major and minor malformations. Both of these two studies were based on retrospective exposure ascertainment. A study from the Swedish Medical Birth Register used maternal reports of thyroxin use in early pregnancy as an exposure indicator and found an increased risk for any malformation and for cardiovascular defects and also high odds ratios, although not statistically significant, for anal atresia and severe kidney malformations [7].

One study used data on thyroxin (T4) level in the newborn as a proxy for early exposure and studied choanal atresia [8]. It found an inverse relationship between malformation risk and T4 level and discussed the possibility that the causal factor was maternal hyperthyroidism or treatment for this condition in early pregnancy.

In the present study we expand the previous study [7] to include pregnancies up to and including 2011.

## 2. Material and Methods

The study was performed using data in the Swedish Medical Birth Register [9] for the years 1996–2011. In this register, information on maternal use of drugs in early pregnancy is recorded, based on midwife interviews performed at the first prenatal care visit, usually in weeks 10–12. The women were asked if they had used any drugs since they became pregnant and the answer was written down in clear text. This information thus basically refers to first trimester use. The drug names were centrally translated to Anatomical, Therapeutic, and Chemical (ATC) codes. The exposure to thyroxin (ATC code H03AA01) was the main exposure studied but some concomitantly used drugs with a possible teratogenic capacity (thyreostatics, anticonvulsants, and drugs used for hypertension) were also identified.

The Medical Birth Register also gave information on some covariates which were used in the analysis: year of delivery, maternal age, parity, smoking habits in early pregnancy, and prepregnancy weight and height from which body mass index (BMI) was calculated. The register also gave delivery diagnoses of preexisting diabetes.

Congenital malformations in the infants were identified from three sources [10]: diagnoses in the Medical Birth Register given by the attending paediatrician, diagnoses reported to the Birth Defect Register (previous Register of Congenital Malformations), and discharge diagnoses from hospitalizations of the children (Patient Register). Linkage between these registers was made using the unique personal identification number everyone living in Sweden has which is widely used in society and in all medical documentation.

Diagnoses of congenital malformations according to the International Classification of Diagnoses (ICD) were codes 740.0–759.9 in ICD-9 (used to 1997) and codes beginning with Q in ICD-10 (used from 1997). In order to eliminate some common and clinically less important conditions with a variable registration, infants with only one or more of the following conditions were excluded: preauricular appendix, tongue tie, patent ductus at preterm birth, single umbilical artery, hip (sub)luxation, undescended testis, and nevus. The remaining conditions were called “relatively severe malformations.”

Risk estimates as odds ratios (ORs) were made with Mantel-Haenszel methodology, adjusting for year of delivery, maternal age (5-year classes, <20, 20–24, etc.), parity (1, 2, 3, and ≥4 where parity 1 = first infant born), maternal smoking in early pregnancy (unknown, none, <10 cigarettes/day, and ≥10 cigarettes/day), and BMI (unknown, <18.5, 18.5–24.9, 25–29.9, 30–34.9, and ≥35). Miettinen's technique was used to determine approximate 95% confidence intervals (95% CI). When the expected number of exposed malformed infants (calculated with adjustment as above) was <10, a risk ratio (RR) was instead calculated as observed/expected numbers with 95% CI from Poisson distributions.

## 3. Results

Table 1 presents characteristics of women who reported the use of thyroxin in early pregnancy, 1.5% of all women giving birth. Women using thyroxin were older than other women giving birth; there was a slightly low use of thyroxin at parity 3 or higher. These women smoked less than other women but had a higher BMI. They had a marked increased risk for diabetes and for using thyreostatics, anticonvulsants, or drugs for hypertension; these drug categories are associated with an increased risk for teratogenesis. For the analysis of congenital malformations in the offspring, these women were therefore excluded.

Among 1 575 847 infants registered in the Medical Birth Register in 1996–2011, 24 622 were born to women reporting substitution with thyroxin in early pregnancy according to the midwife interviews. Exclusion of women with diabetes or using the drugs specified above resulted in 23 259 infants for the analysis of the congenital malformation risk.  Table 2 shows the presence of congenital malformations and the estimated ORs or RRs.

The risk for any relatively severe congenital malformation is only little increased. Before exclusion of women with diabetes or other drugs with a likely teratogenic effect, this OR was 1.11 (95% CI 1.04–1.19). It thus decreased to about half by the exclusion of these women and lost statistical significance, OR = 1.06 (95% CI 0.98–1.14). Among specific malformations, only three showed statistically significant effects: CNS defects other than neural tube defects and choanal and anal atresia. Before exclusion of women with diabetes and so forth, the RR for CNS defects was 1.49 (95% CI 0.97–2.18), for choanal atresia 3.35 (95% CI 1.45–6.60), and for anal atresia 1.97 (95% CI 1.16–2.01). Thus little effects were obtained by exclusion of women with preexisting diabetes and so forth, as corresponding RRs after the exclusions were 1.60 (05% CI 1.05–2.44) for CNS defects, 3.14 (95% CI 1.26–6.47) for choanal atresia, and 1.86 (95% CI 1.00–1.85) for anal atresia.

Among infants with other CNS defects than neural tube defects, five had microcephaly and eight had hydrocephaly (one of them with a cerebral cyst). Six infants had agenesis of corpus callosum (two of them with a cerebral cyst) and one had holoprosencephaly. One infant had a spinal cord malformation and another had an unspecified brain malformation. Agenesis of corpus callosum and holoprosencephaly occurred at an increased rate (RR = 2.54, 95% CI 1.02–5.23).

Increased risk estimates although not reaching statistical significance were seen for oesophageal atresia, abdominal wall defects, diaphragmatic hernia, and severe kidney defects. Among the five infants with abdominal wall defects, three had gastroschisis and two had omphalocele. Among 16 infants with severe kidney malformations, three had agenesis, three had hypoplasia, four had dysplasia, five had polycystic kidneys, and one had an unspecified cystic kidney.

We looked for differences in the risk for a relatively severe malformation in different subgroups of women (age groups, parity groups, and according to smoking and BMI) but no convincing differences were found (data not shown).

## 4. Discussion

Our study had some weaknesses. No information on hypothyroidism in early pregnancy existed but the exposure variable was the reported maternal use of thyroxin. It is likely that the majority of these women on thyroid hormone substitution were euthyroid when they became pregnant. Some women may have taken thyroxin without reporting it but that will hardly affect risk estimates. The absence of clinical details including serum TSH and T4 levels, unavoidable in a study based on register data, makes it impossible to identify subsets of patients which could deviate with respect to the risk for congenital malformations in the offspring. Thus, for instance, the risk could be higher for women with suboptimum thyroxin substitution than in women who were actually euthyroid at the start of the pregnancy.

We have no information on malformed foetuses aborted after prenatal diagnosis. Such foetuses are reported but for legal reasons without identification numbers which makes it impossible to ascertain exposures. This means that an effect on a malformation which is nearly always detected by prenatal diagnosis and nearly always aborted (e.g., anencephaly) will not be detectable but if the malformation is only sometimes detected (e.g., spina bifida), an association can be found but the study power is decreased.

As far as we know this is the largest available study on the subject. We found a weak increase in the risk to have a malformed infant among women who reported the use of thyroxin in early pregnancy but this could be explained by comorbidity with preexisting diabetes. After exclusion of women with that diagnosis (and also women using thyreostatics, anticonvulsants, or drugs for hypertension) a nonsignificant low risk increase remained. It could be due to unidentified cases of diabetes or of the use of thyreostatics, anticonvulsants, or antihypertensives.

This slight and nonsignificant risk increase contrasts strongly with the marked risk described after maternal hypothyroidism [6]; an explanation may be the retrospective ascertainment of exposures in that study and perhaps differences in detection and treatment of this condition in the two study populations. The result of our study is more in line with that by Khoury et al. [5] who found an adjusted OR of 1.07 (95% CI 0.74–1.53) after maternal use of thyroid hormone replacement which is close to the OR in our study. This study was based on only 82 exposed cases, while our study was based on 1092 exposed cases which explains the difference in confidence interval width.

The risk estimate for any malformation in the present study was lower than that found in our previous study [7] in spite of similar methodology: 1.06 versus 1.19. One difference between the studies was that in the present but not in the previous one we took into consideration maternal BMI. In the previous study we found an association with cardiovascular defects which was not quite statistically significant in the present extended study. We found a high RR for severe kidney malformations [7]; it was still increased but had lost its statistical significance. The RR for anal atresia was high and remained high in the present study and now statistical significance was reached. CNS malformations except for neural tube defects or choanal atresia were not studied in the previous investigation.

The finding of a marked and statistically significant risk increase for choanal atresia agrees with the finding presented by Lee et al. [8] in spite of very different exposure criteria. Our study was based on maternal reported thyroxin use in early pregnancy and the Lee et al. study [8] on infant thyroxin level at birth as a proxy of conditions in early pregnancy when choanal atresia will arise. Infant T4 levels were classified by using quartiles based on the distribution among controls. The study was based on 69 cases and 3570 controls; 34 cases had T4 levels in the 1st quartile and five in the 4th quartile. We studied 161 infants with choanal atresia and among them seven were exposed to thyroxin in early pregnancy. In both studies the risk was about three times increased.

It seems unlikely that substitution with thyroxin in itself can cause congenital malformations and as the women took thyroxin, they had probably no clinical hypothyroidism. In the previous paper [7] an explanation was suggested. It was pointed out that the maternal demand of thyroxin increases already in early pregnancy (from week 5) and the embryonic thyroid does not start to produce thyroxin until after gestational weeks 10–12. Therefore, during the period of organogenesis a thyroxin deficiency could exist in the fetus which might interfere with organogenesis. The importance of an adequate replacement therapy with thyroid hormone in early pregnancy in order to avoid suboptimal fetal thyroxin levels is essential. This should be achieved already before the first prenatal care visit which often occurs in weeks 10–12.

To conclude, women who are treated with thyroxin in early pregnancy for hypothyroidism have only a slightly increased risk to have a malformed child and marked effects are only seen on a few relatively rare conditions, CNS malformations except for neural tube defects and choanal and anal atresia.

## Figures and Tables

**Table 1:** Characteristics of women who reported the use of thyroxin in early pregnancy.

Variable	With thyroxin	In population	OR	95% CI
Maternal age				
<20	87	27828	**0.31**	**0.20**–**0.38**
20–24	1331	210254	**0.58**	**0.55–0.62**
25–29	5416	485407	1.00	Reference
30–34	9352	531265	**1.53**	**1.48–1.55**
35–39	6182	248516	**2.10**	**2.02–2.19**
40–44	1560	47018	**2.80**	**2.64–2.98**
≥45	93	2014	**3.66**	**2.99–4.48**
Parity				
1	9247	687283	1.00	Reference
2	9250	562633	1.00	0.97–1.03
3	3696	310094	**0.92**	**0.88–0.96**
≥4	1846	92332	**0.89**	**0.84–0.94**
Smoking				
Unknown	381	95377	—	—
None	22191	1316102	1.00	Reference
<10 cigs/day	1031	100330	**0.87**	**0.72–0.81**
≥10 cigs/day	428	40533	**0.75**	**0.68–0.82**
BMI				
Unknown	1717	192912	—	—
<18.5	349	33624	**0.87**	**0.78–0.97**
18.5–24.9	12112	841302	1.00	Reference
25–29.9	6110	336114	**1.22**	**1.18–1.25**
30–34.9	2509	106013	**1.58**	**1.52–1.65**
≥35	1242	42377	**1.93**	**1.82–2.05**
Maternal diabetes	693	8028	**5.28**	**5.01–5.78**
Maternal use of thyreostatics	173	401	**41.7**	**36.3–47.5**
Maternal use of anticonvulsants	105	4385	**1.38**	**1.14–1.68**
Maternal use of antihypertensives	260	5506	**2.20**	**1.94–2.49**

Total number	24039	1552342	—	—

Odds ratio (OR) with 95% confidence intervals (95% CI) after adjustment for year of birth and maternal age, parity, smoking, and BMI. Bold figures mark statistical significance.

**Table 2:** Presence of congenital malformations in infants whose mothers reported the use of thyroxin in early pregnancy (*n* = 23 259) and in all infants born (*n* = 1 567 736).

Malformation	Number with thyroxin	Total number	OR/RR	95% CI
Any relatively severe malformation	730	48 012	1.06	0.98–1.14
Any chromosome anomaly	59	2927	1.18	0.91–1.53
Down syndrome	42	1911	1.25	0.92–1.69
Excluding chromosome anomalies				
Neural tube defects	7	725	0.81	0.33–1.68^#^
Other CNS malformations	22	1118	**1.60**	**1.05**–**2.44**
Severe eye malformations	6	577	0.84	0.34–1.74^#^
Severe ear malformations	4	277	1.03	0.28–2.63^#^
Choanal atresia	7	161	**3.14**	1.26–6.47^#^
Orofacial clefts	40	2740	1.01	0.79–1.38
Cardiovascular defects	245	15891	1.05	0.92–1.19
Septal defects	171	10974	1.04	0.94–1.28
Oesophageal atresia	9	444	1.38	0.63–2.62^#^
Small gut atresia	3	390	0.54	0.11–1.58^#^
Anal atresia	16	588	**1.85**	1.00–1.85^#^
Pyloric stenosis	11	1095	0.74	0.41–1.33
Abdominal wall defects	5	412	1.28	0.42–2.98^#^
Diaphragmatic hernia	8	365	1.56	0.67–3.08^#^
Hypospadias	70	4508	1.05	0.82–1.32
Severe kidney malformations	16	873	1.32	0.81–2.17
Pes equinovarus	28	2115	0.93	0.64–1.35
Poly- or syndactyly	38	3065	0.93	0.60–1.14
Limb reduction defects	13	826	1.13	0.65–1.96
Craniosynostosis	10	857	0.79	0.42–1.48

Odds ratio (OR) or risk ratio (RR marked^#^) with 95% confidence intervals (95% CI). Bold text marks statistical significance.
